# Self-learning activation functions to increase accuracy of privacy-preserving Convolutional Neural Networks with homomorphic encryption

**DOI:** 10.1371/journal.pone.0306420

**Published:** 2024-07-22

**Authors:** Bernardo Pulido-Gaytan, Andrei Tchernykh

**Affiliations:** 1 Computer Science Department, CICESE Research Center, Ensenada, BC, Mexico; 2 Ivannikov Institute for System Programming, RAS, Moscow, Russia; Manipal Institute of Technology, Manipal Academy of Higher Education, INDIA

## Abstract

The widespread adoption of cloud computing necessitates privacy-preserving techniques that allow information to be processed without disclosure. This paper proposes a method to increase the accuracy and performance of privacy-preserving Convolutional Neural Networks with Homomorphic Encryption (CNN-HE) by Self-Learning Activation Functions (SLAF). SLAFs are polynomials with trainable coefficients updated during training, together with synaptic weights, for each polynomial independently to learn task-specific and CNN-specific features. We theoretically prove its feasibility to approximate any continuous activation function to the desired error as a function of the SLAF degree. Two CNN-HE models are proposed: CNN-HE-SLAF and CNN-HE-SLAF-R. In the first model, all activation functions are replaced by SLAFs, and CNN is trained to find weights and coefficients. In the second one, CNN is trained with the original activation, then weights are fixed, activation is substituted by SLAF, and CNN is shortly re-trained to adapt SLAF coefficients. We show that such self-learning can achieve the same accuracy 99.38% as a non-polynomial ReLU over non-homomorphic CNNs and lead to an increase in accuracy (99.21%) and higher performance (6.26 times faster) than the state-of-the-art CNN-HE CryptoNets on the MNIST optical character recognition benchmark dataset.

## 1. Introduction

Machine Learning (ML) solutions based on Neural Networks (NN) are extensively used in multiple domains due to the remarkable results achieved in image rendering [1], business decision-making [2], pharmaceutical [3], bioinformatics [4], cancer research [5], and drug discovery, among many others. However, NN modeling demands considerable infrastructure and computing power for calculations and training with big data in a reasonable amount of time [6].

Cloud computing provides the necessary infrastructure and services to implement, train, and deploy NN models [7, 8]. However, using NNs on third-party infrastructures reveals several data privacy issues during the pre-processing, processing, and post-processing [9]. Third-party infrastructure reduces resource costs and makes it easier to solve tasks but introduces privacy issues of sensitive information processing [10, 11]. The access of the NN to the raw data can create potential privacy risks because data are located in the cloud-shared infrastructure. In general, cloud environments provide cost-savings, flexibility, scalability, and ubiquitous computing resources, but they potentially sacrifice the privacy of information [12]. Their main constraints are insufficient protection of sensitive data processing and the inherently insecure nature of shared resources.

Modern encryption techniques ensure security and are considered the best option to protect stored data and data in transit from an unauthorized third party. However, a decryption process is necessary when the data must be processed or analyzed, falling into the initial problem of data vulnerability. Efficient processing of sensitive data implies beyond an accurate prediction, analysis, or classification; it also implies adequate privacy handling.

Homomorphic Encryption (HE) schemes are solutions to address privacy problems by providing encrypted data computations to the client [13, 14]. Applications with HE provide security and confidentiality to users, even when they are executed on untrusted shared infrastructures. However, HE schemes have limitations concerning the arithmetic operations that can be executed efficiently. They are bound to solve real problems only using additions and multiplications.

Recent advances in HE focus on incorporating HE cryptosystems into NN models. However, redesigning neuron functions and developing HE-friendly non-linear operations like comparison, max/min, sign detection, etc. [15] are open problems.

Activation functions like Rectified Linear Unit (ReLU), Leaky ReLU, Parametric ReLU, Gaussian Error Linear Unit (GELU), Sigmoid, Tangent Hyperbolic (Tanh), Softmax, Swish, Binary Step, etc. are non-polynomial and use operations not supported by HE schemes. Therefore, the efficient implementation of cryptographically computable activation functions becomes imperative for developing a privacy-preserving NN with HE (NN-HE) [16].

Several polynomial approximation methods are used to support homomorphic computation in NN-HE, such as Taylor series, Least-squares, Chebyshev series, Newton, Composite polynomials, etc.

Another important aspect is to allow homomorphic computations in the domain of continuous values. The Cheon-Kim-Kim-Song (CKKS) scheme [17] is designed to perform approximate arithmetic over encrypted real numbers. While the CKKS scheme uses state-of-the-art solutions to support approximations, they are still inefficient in practice, and thus, efficient HE-compliant activation functions remain an open challenge [18].

Several limitations arise in reproducing the behavior of neurons using polynomial approximations in the homomorphic space [19]. The main goal is to control the trade-off between having a non-linear transformation, which is needed by the learning algorithm, and keeping the degree of the polynomials small to make HE feasible [20–22].

In this paper, we design an adaptive NN-HE with improved accuracy and performance. Optimization is supported by polynomial Self-Learning Activation Functions (SLAF). Trainable coefficients of polynomial approximations of NN activation functions are updated during the training together with synaptic weights at each neuron independently to learn problem-specific, dataset-specific, and NN-specific features.

Two Convolutional Neural Network (CNN) models are proposed: CNN-HE-SLAF and CNN-HE-SLAF-R. In the first model, all the activation functions are replaced with SLAFs, and CNN is trained to find weights and coefficients. In the second one, CNN is trained with the original activation function, then the found weights are fixed, activation functions are substituted by polynomials, and CNN is shortly re-trained to adapt SLAF coefficients at each polynomial.

We present two SLAF initialization approaches: SLAF_(0)_ and SLAF_(P)_. The SLAF_(0)_ considers trainable polynomial coefficients initialized to zero. In SLAF_(P)_, coefficients are initialized by values of known polynomial approximations.

CNN-HE-SLAF and CNN-HE-SLAF-R are non-interactive privacy-preserving models to classify encrypted data. For a detailed assessment of the capabilities of the SLAF approach, we conduct a comprehensive analysis over 1-convolutional (CNN1) and 2-convolutional (CNN2) NNs using both plaintext and ciphertext as inputs from the MNIST dataset.

These results show the remarkable capability of HE-SLAF to yield comparable accuracy to traditional activation functions, indicating that models with trainable polynomial activations have the same representative ability as their non-polynomial counterparts. HE-SLAF low-degree polynomials achieve the same accuracy as a non-polynomial ReLU activation function over non-homomorphic models in the plaintext space.

On the other hand, we show that NN-HE-SLAF models with trainable three-degree polynomial activations improve accuracy over known NN-HE solutions in the literature. The training of polynomial coefficients of each neuron independently allows the exploration of the space of all the activations to construct better homomorphically evaluated approximations.

The content of the paper is structured as follows. Section 2 introduces privacy-preserving techniques, delving into homomorphic encryption schemes. Section 3 presents the primitives of the CKKS scheme. Section 4 describes privacy-preserving neural networks with homomorphic encryption. Section 5 discusses state-of-the-art NN-HE models that polynomially approximate cryptographically non-computable activation functions. Section 6 presents related work. Section 7 introduces homomorphic self-learning activation functions. Section 8 provides a brief overview of the case study. Section 9 defines the experimental setup. The experimental results are presented in Section 10. Finally, we discuss our solutions and conclude in Section 11.

To facilitate understanding of the described ideas, we summarize the main HE, NN, and SLAF terminology in the Appendix. S1 Table shows the terms used in the paper, and S2 Table presents the main acronyms.

## 2. Privacy-preserving techniques

Privacy-preserving methods are emerging as a crucial consideration in cloud environments. Secure Multi-Party Computation (MPC) [23, 24], Federated Learning (FL) [25, 26], Differential Privacy (DP) [27], Functional Encryption (FE) [28], and Homomorphic Encryption (HE) are representative approaches in this domain.

### 2.1. Secure multi-party computation

Multi-Party Computation (MPC) encompasses several cryptographic algorithms and protocols that allow multiple mutually distrustful parties to calculate jointly a function over their inputs while keeping those inputs private: garbled circuits, secret sharing schemes, and oblivious transfer.

In MPC, regardless of dishonest behavior and information sharing among some colluded parties, they should not be able to learn more about the other participants’ inputs beyond what can be inferred from the computation result. MPC guarantees that even with such a collusion, the privacy and correctness of the collaborative computation are preserved.

Formally, *n* parties with their respective private data *d*_1_, *d*_2_,…,*d*_*n*_ can jointly compute the public function *f*(*d*_1_, *d*_2_,…,*d*_*n*_) while preserving their input secret. The parties compute *f* on *d*_1_, *d*_2_,…,*d*_*n*_ without compromising confidentiality. The information about parties’ secrets must not be leaked during the process.

Yao’s work [24] proposed the concept of secure two-party computation by inventing the garbled circuit. The subsequent extension to MPC is provided by Goldreich et al. [23]. Several methods were proposed to evaluate privacy-preserving Neural Network (NN) models using MPC protocols. However, these protocols imply a high communication overhead, making the latency of models a problem. Communication costs can be minimized by performing resource-intensive pre-computations, such as the NN non-linear activations, during an offline phase or using trusted execution environments [20].

### 2.2. Federated learning

Federated Learning (FL) is an approach to train NN models across multiple decentralized parties holding local data samples. It allows the model training without explicitly exchanging the data between parties, guaranteeing privacy while enabling collaborative learning.

The FL process involves training local models on each party, which are then aggregated into a comprehensive global model. The gradient updates, rather than the data itself, are shared across the network. Subsequently, a copy of the global model is disseminated to each party to repeat the process. This procedure iterates until the model performance meets the desired criteria.

Although FL does not inherently provide cryptographic privacy guarantees [25], gradient aggregation can be secured using privacy-preserving techniques such as MPC and HE [26]. FL is a prominent technique in scenarios that require strict respect for data confidentiality, e.g., where data dissemination is restricted due to regulatory constraints or to protect individual privacy. The main FL drawbacks are the need for a trusted gradient aggregation manager and communication overhead.

### 2.3. Differential privacy

Differential Privacy (DP) is a mathematical framework that adds random noise to data to provide privacy guarantees for individual-level information while enabling accurate aggregate analysis. It quantifies the anonymization of sensitive data based on the amount of noise added.

A randomized function *δ* provides *ϵ*-differential privacy if, for all datasets *d*_1_ and *d*_2_ differing on at most one element, and all *S*⊆*Range*(*δ*) [27], it holds that

$$Pr[\delta (d_{1})\in S]\geq \exp\;\epsilon \times Pr[\delta (d_{2})\in S]$$ (1)

where *ϵ* denotes the privacy level. Smaller *ϵ* values indicate higher privacy levels. Eq (1) implies that the probability of *δ* producing an output in the set *S* for *d*_1_ should be no less than exp *ϵ* times the probability of *δ* producing an output in *S* for *d*_2_.

Therefore, the computation output remains statistically indistinguishable, within parameter *ϵ*, when performed on any two datasets that differ by a single individual’s data. This statistical property makes it challenging for an adversary to extract specific information about any individual from the function output, thereby preserving individual-level privacy [29].

Within the scope of NN models, DP prevents the disclosure of individual information in training. The aim is to discourage attempts of data extraction from the trained model by controlling the impact of each sample on the parameters during training. FL can also employ DP to collaboratively train a shared model on decentralized data without revealing inherent information about data to other participants. However, the injected noise leads to a decrease in model accuracy. Furthermore, DP only offers a probabilistic bound on potential information leakage rather than a cryptographic privacy guarantee [30].

### 2.4. Functional encryption

Functional Encryption (FE) is a cryptographic scheme that supports a controlled functional evaluation of encrypted data without plaintext access. In FE, the result of function evaluation is directly revealed in the decryption process [28].

More precisely, in an FE system for a function *f*, a user generates a ciphertext *c* from a plaintext *m* using a public key *pk*. A third party, known as the decryptor, who possesses a decryption key *dk*_*f*_ for *f* (issued by a trusted manager holding secret key *sk*) can compute *f*(*m*) by decrypting *c*. In other words, FE performs a specific *f* on *c* without revealing the underlying information *m* to the decryptor.

Incorporating FE schemes into privacy-preserving NN models implies that only the initial model layer is computed on encrypted data. In contrast, the subsequent hidden layers undergo evaluation utilizing plaintext data, potentially leaking the intermediate computation results and the model output to the decryptor entity.

### 2.5. Homomorphic encryption

In cryptography, the term Homomorphic Encryption (HE) describes a form of encryption capable of performing certain operations over ciphertexts without access to the secret key. The information is public without representing a risk of a data breach. The output of the calculated functions remains encrypted. Its correctness relies on the homomorphism concept, a structure-preserving transformation where two groups in different spaces can be mapped. In this case, a homomorphic function applied to ciphertexts provides the same result (after decryption) as using the function to the original unencrypted data.

Unlike MPC and DP privacy-preserving techniques, third-party systems with HE protect the entire data lifecycle (data storage, data transmission, and data processing) without needing trusted data managers and multiple rounds of client-server communication. HE enables blind, two-party non-interactive processing of sensitive data [13].

Let *m*_*a*_ be the message *a* in plaintext, *sk* a secret key for decryption, and *pk* a public key for encryption. The corresponding ciphertext *c*_*a*_ of *m*_*a*_ is generated by the encryption operation *c*_*a*_ = *Encrypt*(*pk*, *m*_*a*_).

Recovering the information in an additively HE from a ciphertext *c*_+_ is performed by the decryption operation and the secret key as *m*_+_ = *Decrypt*(*sk*, *c*_+_), where *c*_+_ = *Add*(*c*_*a*_, *c*_*b*_) contains the result of the homomorphic addition between *c*_*a*_ and *c*_*b*_. Analogously for multiplication *c*_×_ = *Mult*(*c*_*a*_, *c*_*b*_) in a multiplicative HE. The HE schemes obtain ciphertexts *c*_+_ and *c*_×_, without knowing *m*_1_ and *m*_2_. Ciphertexts *c*_+_ and *c*_×_ cannot be computed with conventional encryption without the decryption of *c*_*a*_ and *c*_*b*_.

Each HE scheme defines a conventional public-key scheme with basic operations to generate secret and public keys, encrypt and decrypt ciphertexts, and perform homomorphic additions and multiplications [31].

Several HE schemes are proposed. They include Brakerski-Gentry-Vaikuntanathan (BGV) [32], Brakerski/Fan-Vercauteren (BFV) [33, 34], Gentry-Sahai-Waters (GSW) [14], YASHE [35], Hoffstein-Pipher-Silverman (HPS) [36], López-Tromer-Vaikuntanathan (LTV) [37], and Cheon-Kim-Kim-Song (CKKS) [17] schemes. All of them pursue the implementation of more efficient HE schemes.

A distinctive feature of CKKS is the implementation of approximate arithmetic over encrypted real (complex) numbers, thereby allowing for privacy-preserving computations on sensitive data in the domain of continuous values. This capability of CKKS is crucial for complex and demanding applications, such as privacy-preserving NN [16, 38].

The following section describes the CKKS scheme primitives used throughout this manuscript as the underlying HE cryptosystem.

## 3. CKKS homomorphic encryption scheme

CKKS is a lattice-based HE scheme whose security is based on the hardness of the Ring Learning with Errors (RLWE) problem. The CKKS scheme performs approximate arithmetic over encrypted real (complex) numbers. Given messages *m*_1_ and *m*_2_, it allows secure computing encryptions of approximate values of *m*_1_+*m*_2_ and *m*_1_*m*_2_ with a prefixed precision. The main characteristic of CKKS is that it treats the inserted noise of the RLWE problem as part of an error occurring during approximate computation.

In CKKS, after the selection of parameters such as degree *N* of the polynomial ring and a modulo *q*, real (complex) numbers are encoded into plaintext polynomials. The secret key is a randomly generated polynomial. The public key is created using the secret key, chosen parameters, and some randomness. The plaintext polynomial is encrypted using the public key and noise to increase security. After operations, rescaling is performed to reduce noise and preserve the ciphertext. The ciphertext is decrypted using the secret key to obtain an approximated version of the original plaintext polynomial that is decoded into a real or complex resulting number.

Let the polynomial ring for a power-of-two *N* be *R* = ℤ[*X*]/(*X*^*N*^+1), and *R*_*q*_ = ℤ_*q*_[*X*]/(*X*^*N*^+1) be a modulo-*q* residue ring of *R*. The polynomial coefficients of *R*_*q*_ are bounded by the modulo *q* and the degree of polynomials by *X*^*N*^+1. Let *L* be a level parameter that indicates the maximum multiplicative depth. Modulo *q* = *q*_0_∙*q*_1_∙…∙*q*_*L*_ is defined as the product of co-prime moduli *q*_0_,…*q*_*L*_, where *q*_ℓ_ = 2^ℓ^∙*q*_0_ for 1≤ℓ≤*L*.

The distribution *χ*_key_ = HW(*h*) outputs a polynomial from *R*_*q*_ of {±} -coefficient having *h* non-zero coefficients, where HW(*h*) denotes the set of signed binary vectors in {±}^*N*^ whose Hamming weight is *h*∈*Z*_+_. *χ*_enc_ and *χ*_err_ denote discrete Gaussian distributions with some predefined standard deviation. *U*(*R*_*q*_) refers to a uniform distribution over the ring *R*_*q*_. Moreover, for $a=\sum_{i=0}^{N-1}a_{i}X^{i}\in R[X]/(X^{N}+1)$, then $\left\lfloor a\right\rceil =\sum_{i=0}^{N-1}\left\lfloor a_{i}\right\rceil X^{i}\in R$, where ⌊∙⌉ returns the nearest integer of a real-number input, rounding upwards in case of a tie [17].

CKKS encodes real values using what is known as canonical embedding $\tau :\;R[X]/(X^{N}+1)\to C^{N/2}$, where a plaintext vector $\vec{m}=(m_{0},\ldots ,m_{N/2})$ is transformed into $\tau^{-1}(\vec{m})\in R[X]/(X^{N}+1)$ and then rounded to an integer-coefficient polynomial using a scaling factor Δ, i.e., $\left\lfloor \Delta ∙\tau^{-1}(\vec{m})\right\rceil$. The parameter Δ affects the accuracy of the computation in CKKS.

Let us discuss the main primitives of the CKKS scheme: *KeyGen*, *Encrypt*, *Decrypt*, *Add*, *Mult*, *Rot*, and *Resc*.

- *KeyGen* (*N*, *q*, *L*)→*sk*, *pk*, *ek*. Sets secret key as *sk* = (1,*s*), where *s*←*χ*_key_. Sets public key as $pk=(b,a)\in R_{q_{L}}^{2}$, where *b* = −*as*+*e* (mod *q*_*L*_), $a\leftarrow U(R_{q_{L}})$, and *e*←*χ*_err_. The evaluation key is set as $ek=(b^{\prime },a\prime )\in R_{q_{L}^{2}}^{2}$, where $b^{\prime }=-a^{\prime }s+e^{\prime }+q_{L}s^{2}(mod\;q_{L}^{2})$, $a\prime \leftarrow U(R_{q_{L}^{2}})$ and *e*′←*χ*_err_.
- $Encrypt(\vec{m},\Delta ,pk)\to c$. For a plaintext vector of real (complex) numbers $\vec{m}$, ist encodes $m=\left\lfloor \Delta ∙\tau^{-1}(\vec{m})\right\rceil \in R$, and provides the ciphertext $c=v∙pk+(m+e_{0},e_{1})(mod\;q_{L})$, where *v*←*χ*_enc_ and *e*_0_, *e*_1_←*χ*_err_.
- $Decrypt(c,\Delta ,sk)\to \vec{m}$: For a ciphertext $c=(c_{0},c_{1})\in R_{q_{l}}^{2}$, decodes the message as $m=c_{0}+c_{1}∙s(mod\;q_{l})$, and outputs a plaintext vector $\vec{m}=\Delta^{-1}∙\tau (m)$.
- *Add*(*c*_1_, *c*_2_)→*c*_+_: Add two ciphertexts $c_{1},c_{2}\in R_{q_{l}}^{2}$. It returns ciphertext $c_{+}=c_{1}⊕c_{2}=c_{1}+c_{2}(mod\;q_{l})$.
- $Mult(c_{1},c_{2},ek)\to c_{\times }$: For two ciphertexts $c_{1}=(c_{1,0},c_{1,1}),c_{2}=(c_{2,0},c_{2,1})\in R_{q_{l}}^{2}$, let $\left(d_{0},d_{1},d_{2})=(c_{1,0}c_{2,0},c_{1,0}c_{2,1}+c_{1,1}c_{2,0},c_{1,1}c_{2,1}\right)$. It returns ciphertext $c_{\times }=c_{1}⊗c_{2}=(d_{0},d_{1})+\left\lfloor q_{L}^{-1}∙d_{2}∙ek\right\rceil (mod\;q_{l})$.
- *Rot*(*c*,*r*)→*c*′: Given an encryption *c* of $\vec{m}=(m_{0},\ldots ,m_{N/2})$, outputs *c*′ that encrypts the left-rotated vector $\vec{m}=(m_{r},\ldots ,m_{N/2},m_{0},\ldots ,m_{r-1})$ by *r* positions.
- Since every $\vec{m}$ is scaled, the plaintext of *c*←*Mult*(*c*_1_, *c*_2_) is Δ∙*m*_1_*m*_2_, which results in an exponential growth of plaintexts. To deal with such a problem, CKKS introduces the so-called rescaling procedure:
- *Resc*(*c*)→*c*′: For a ciphertext $c\in R_{q_{l}}^{2}$, outputs $c^{\prime }=\left\lfloor q_{l^{\prime }}/q_{l}\right\rceil ∙c(mod\;q_{l^{\prime }})$.

For more detailed information and additional considerations on the CKKS scheme, including the correctness and security analysis, refer to [17].

## 4. Privacy-preserving NN-HE

This section focuses on understanding the construction and evaluation of the privacy-preserving NN model with HE (NN-HE).

NN-HE is a natural extension of NN models. It consists of applying HE to the network inputs and homomorphically propagating the data across the network. NN-HE implementation has several restrictions because not all the functions for processing NN have homomorphic counterparts. So, the internal NN structure must be modified to perform secure processing. One way is to substitute non-homomorphic operations with appropriate approximations.

The main challenge in constructing NN-HE models is designing the efficient homomorphic processing of inner network functions. In the following sections, we discuss this complex problem, considering homomorphic neurons and homomorphic layers.

### 4.1. Homomorphic neuron

Homomorphic neurons are the processing that involves several operations. Each neuron performs a weighted-sum and a non-linear activation function defined by

$$y\leftarrow f(\sum_{i=1}^{n}(w_{i}\times x_{i})+\beta )$$ (2)

where *n* denotes the number of neuron inputs *x*_*i*_ with weights *w*_*i*_, for *i* = 1…*n*. The weighted-sum consists of additions and multiplications between the inputs and their corresponding synaptic weights, enabling its computation over encrypted data.

The most important and still open problem in the NN-HE is the definition of adequate non-linear components. Standard activation functions, such as Rectified Linear Unit (ReLU), are not polynomials and use operations not supported by HE schemes. So, finding cryptographically compatible replacement functions is imperative to developing privacy-preserving NN-HE models [39].

The sign function can be used to implement ReLU over fixed-precision numbers [40]. The ReLU function is denoted as

$$ReLU(x)=\frac{x+x∙sign(x)}{2}=\left\{ \begin{array}{c} x\;if\;x>0,\\ 0\;if\;x\leq 0, \end{array}\right.$$ (3)

where *sign*(*x*) function is defined by

$$sign(x)=\left\{ \begin{array}{l} \;1\;if\;x>0,\\ \;0\;if\;x=0,\\ -1\;if\;x<0. \end{array}\right.$$ (4)

An accurate polynomial approximation of *sign*(*x*) is enough to perform a HE-compliant ReLU operation [41]. It considers two intervals [−1, −*ϵ*]∪[*ϵ*, 1] because *sign*(*x*) is discontinuous at *x* = 0.

Therefore, the homomorphic neuron is defined by

$$\bar{y}\leftarrow \ddot{f}(\sum_{i=1}^{n}({\bar{w}}_{i}\ddot{\times }c_{i})\ddot{+}\bar{\beta }),$$ (5)

where *n* denotes the number of encrypted inputs *c* with weights $\bar{w}$. Bias $\bar{\beta }$ is a ciphertext, and the activation function $\ddot{f}$ is a polynomial approximation that only consists of homomorphic operations $\ddot{+}$ and $\ddot{\times }.\bar{y}$ is the encrypted output of the neuron. It guarantees the privacy of the result even if $\bar{y}$ is disclosed.

Several studies have been conducted to implement the sign function efficiently in the CKKS scheme. These approaches approximate the sign function through Taylor series, least-squares, Newton-Raphson, Fourier series, Chebyshev polynomials, and composite polynomials, among others [39, 40, 42–51]. However, state-of-the-art solutions are still inefficient in practice, and thus, implementing an efficient homomorphically computable activation remains an open challenge [52].

The main goal is to control the trade-off between having a non-linear transformation needed by the learning algorithm and keeping the degree of the polynomials small to make HE parameters feasible. The polynomial degree is directly related to the desired error in a given interval. As the degree grows, the lower approximation error is guaranteed and, thus, better precision. However, a higher computational cost is required.

### 4.2. Homomorphic NN layers

The architecture of the NN defines a set of neurons organized in layers, with connections between them. This section details the homomorphic processing of the standard layers in NN-HEs and their applicability to ciphertexts: fully-connected, convolutional, batch normalization, and pooling layers. The encrypted data adds complexity to the design of NN-HE architectures, which must meet the HE constraints.

A *fully connected layer*, also known as a dense layer, connects each neuron in the current layer to every neuron in the previous layer. It is the most basic and commonly used type of layer in NN architectures. The connection between two consecutive dense layers is represented as a complete graph where each edge has an associated weight. The operation in a neuron is a dot product between its inputs (outputs from the neurons of the previous layer) and their weights. This dot product consists of multiplication and addition operations, enabling its computation over HE-encrypted data. Dense layers enable complex interactions, providing ample opportunity to identify complex patterns and relationships among the data. However, without an effective regularization method, they lead to increased computational complexity and potential overfitting when NN tries to learn too many details in the training data, resulting in poor evaluation.

A *convolutional layer* is the fundamental building block of Convolutional Neural Network (CNN) models, designed to adaptively learn spatial hierarchies of features from input data [53]. It applies a set of trainable filters, also known as kernels, to the input data. Each filter is responsible for extracting a specific feature from data. The filter is moved across the input data with a specific stride, computing the dot product between the filter weights and input values for each spatial position. This process generates a feature map, also known as an activation map, representing the locations and strengths of the learned features within the input data.

Multiple filters can be applied, creating a stack of feature maps as output from the layer. This operation is especially effective for image processing, as it can capture spatial features like edges, corners, and textures while reducing complexity by sharing weights across space. As a result, a convolutional layer implies matrix multiplications and dot products, which are HE-compliant since they only consist of additions and multiplications. Hence, this layer does not need to be modified to process data homomorphically. The substitution of operations by their homomorphic versions is enough to use it.

Several studies have been conducted to speed up convolutional NN-HEs by reducing the overall number of arithmetic operations through techniques such as ciphertext packing and Single Instruction Multiple Data (SIMD) [19] operations.

The *pooling layer* is a sampling layer that reduces the spatial dimensions of the input while retaining important features. It allows the subsequent layers to focus on higher-level representations and makes the model more efficient and invariant to specific spatial transformations.

The pooling operates on each feature map independently by moving a non-overlapping window across the data. There are two main pooling layers: max and average. Max pooling operation searches for the maximum value (strongest response) in the window, making it inefficient when dealing with encrypted data. In contrast, average pooling computes the average value within each region. As a result, average pooling is the solution to be implemented in NN-HE models [19, 47], where the division operation is substituted with multiplication by a constant.

The *batch normalization* (BN) layer stabilizes and accelerates the training process by addressing the internal covariate shift phenomenon caused by continuous changes in the distribution of each layer’s inputs during training. BN makes normalization a part of the model architecture and performs it for each training mini-batch. The BN layer forces the inputs to follow a normal distribution with zero mean and unit variance. In addition to accelerating training by allowing higher learning rates without the risk of divergence, it offers additional indirect benefits for NN-HE models. For instance, a BN layer incorporated before a polynomial activation encourages the pre-activation values to fit in the approximated interval, which reduces the overall approximation error, prevents the generalization of higher feature values, and indirectly provides smaller weights for homomorphic processing. It helps mitigate the potential overflow of values beyond the bounds of the plaintext space.

## 5. State-of-the-art NN-HE models

This section presents the state-of-the-art privacy-preserving NN-HE models, highlighting their implementation details, latency (Lat), and accuracy (Acc).

Table 1 summarizes the essential characteristics of the NN-HEs. The “Layers” column corresponds to the number of convolutional, dense, activation, and pooling layers incorporated within each NN-HE architecture. GPU (Graphic Processing Unit) and 2-arch (2-architecture) columns denote specific aspects of the model’s operation. GPU indicates that the model takes advantage of hardware acceleration methodologies through GPUs. 2-architecture indicates the use of a dual-architecture strategy by collapsing adjacent linear layers during the evaluation process.

**Table 1:** State-of-the-art privacy-preserving neural networks with homomorphic encryption.

Year	Model	Dataset	Performance		Layers				GPU	2-arch	Ref
			Lat (s)	Acc (%)	Conv	Dense	Act	Pool			
2016	CryptoNets	MNIST	250	98.95	2	2	2	2		●	[54]
2017	Chabanne-NN	MNIST	NR*	97.95	2	2	1	3			[47]
			NR*	99.28	6	2	6	2			
2018	F-CryptoNets	MNIST	39.1	98.70	2	2	2	2			[44]
		CIFAR-10	22372	76.72	6	2	3	2			
2018	FHE-DiNN100	MNIST	1.65	96.35	0	1	1	0			[46]
2018	TAPAS	MNIST	37 [hrs.]	98.60	6	2	8	3			[55]
2019	SEALion	MNIST	60	98.91	2	2	1	1			[56]
2019	CryptoDL	MNIST	148.97	98.52	2	2	1	1			[42]
			320	99.25	6	2	3	2			
2019	Lo-La	MNIST	0.29	96.92	1	1	1	0			[57]
			2.20	98.95	2	2	2	2		●	
		CIFAR-10	730	74.10	3	2	2	3			
2019	nGraph-HE	MNIST	16.72	98.95	2	2	2	2		●	[58]
		CIFAR-10	1651	62.20	2	1	2	1			
2019	E2DM	MNIST	1.69	98.10	2	2	2	2		●	[59]
2020	HCNN	MNIST	5.16	99.00	2	1	2	0	●		[19]
		CIFAR-10	304.43	77.55	3	2	3	3			
2022	LeNet-HE	MNIST	138	98.18	2	1	1	2			[60]
2022	RNS-CKKS-NN	CIFAR-10	10602	92.43**	18	1	19	1	●		[50]
2023	CNN-HE-SLAF***	MNIST	3.13	98.22	1	2	2	0		●	
			39.84	99.21	2	2	2	2			

NR*: They do not provide results based on encrypted data. Therefore, we cannot provide a comparison with the performance measures.

**: Classification accuracy with 383 encrypted images.

***: CNN-HE-SLAF is the proposed model.

Dowlin et al. [54] propose CryptoNets to address the challenge of achieving a blind non-interactive classification. The approach uses the leveled YASHE [35] scheme for NN inputs and propagates signals across the network homomorphically.

CryptoNets contains two convolutional layers and two dense layers. It replaces the activation function with the square function, the lowest-degree non-linear polynomial function. Its performance is limited due to using the square function as an activation function, computational overhead, and the insecure YASHE scheme [61]. Several subsequent works in the literature focus on improving its constraints.

Chabanne et al. [47] present the premises of a privacy-preserving Deep Neural Network (DNN) evaluated homomorphically. To improve CryptoNets’ performance, the authors polynomially approximate the ReLU activation function and use a BN layer before each activation layer; the normalization layer limits the need for an accurate approximation to a small part of ℝ around the neighborhood of zero point. However, although the approach indicates the use of the BGV HE scheme, it does not provide any results on the encrypted data.

Chou et al. [44] introduce pruning and quantization methods that leverage sparse representations in the underlying BFV cryptosystem to accelerate CryptoNets inference. Faster-CryptoNets iteratively removes and clusters the model parameters without affecting accuracy. They derive a quantized minimax approximation for standard activation functions that achieves maximally-sparse encodings and optimizes approximation error [62]. Experiments over MNIST show that the NN-HE model maintains competitive accuracy and achieves a significant speedup over previous methods. On CIFAR-10, the proposed model takes more than 6 hours and achieves an accuracy of 76.7%. Also, the authors propose a transfer learning approach with DP to address real-world tasks like medical imaging applications.

Bourse et al. [46] implement a Discretized Neural Network (DiNN) for inference over encrypted data, whose complexity is linear in the network size. Weights and inputs are discretized into elements in [−1,1] with a threshold value equal to 128: any pixel whose value is smaller than the threshold is mapped to −1, otherwise to +1. Although this method can homomorphically evaluate NNs of any depth, each neuron output is refreshed through the bootstrapping procedure featured by the Fully Homomorphic Encryption scheme over the Torus (TFHE), resulting in high overhead and low accuracy. They construct two simple FHE-DiNN models with one hidden (dense) layer containing 30 and 100 neurons with a security level of 80 bits.

Sanyal et al. [55] propose a privacy-preserving Binary Neural Network (BNN) with a TFHE scheme built upon the FHE-DiNN approach. The BNN evaluates every arithmetic operation as a composition of binary gates; it performs homomorphic multiplication by applying the logical operator XNOR and homomorphic addition by summing the number of 1s. TAPAS implements sparsification techniques and algorithmic tools to speed up and parallelize ciphertext computation. Every operation consists of many bootstrapping procedures, is therefore inherently immune to noise, and has no architectural restrictions. Nonetheless, the model is slower than any previous work: one prediction on MNIST takes 37 hours on a single machine and 2.41 hours using a cluster of 16 machines with 16 cores each.

Van Elsloo et al. [56] propose SEALion, an extensible framework built upon the CryptoNets approach for implementing privacy-preserving NN-HE models. SEALion performs an automatic encryption parameter selection for BFV, which side-steps complex implementation details for ad-hoc homomorphic solutions. The proposed approach improves both latency and encrypted inference by sparsifying the activation functions with the *L*_0_ norm.

Hesamifard et al. [42] develop an NN-HE based on the model architecture of [47, 54]. CryptoDL replaces standard non-linear activation functions with HE-friendly low-degree polynomial approximations within a specific error range. ReLU, Sigmoid, and Tangent Hyperbolic (Tanh) functions are approximated using Chebyshev and Taylor series. Additionally, the authors implement a scaled-up version of average pooling, which calculates the summation of values without dividing by the number of values. Building on this approach, Liao et al. [63] implement a CryptoDL-based NN-HE model over encrypted sensor data; the authors approximate Tanh, ReLU, and Swish functions to generate cryptographically computable activations.

Brutzkus et al. [57] propose Low-Latency (Lo-La) CryptoNets to improve latency and memory usage over its predecessors. While CryptoNets encodes each image’s feature as a separate message, Lo-La encrypts entire layers as a single message with the BFV scheme and uses different matrix-vector multiplication implementations throughout the inference. These technical modifications allowed the evaluation of the same model as CryptoNets in a pair of seconds and the private inference over larger datasets such as CIFAR-10. However, the scheme is still dependent on the message dimension and, therefore, impractical for applications with high-dimension data. Additionally, they present the premises of using transfer learning to solve HE limitations, such as message size and noise growth.

Boemer et al. [58] introduce nGraph-HE, an extension of the Intel nGraph compiler to deploy NN models on homomorphically encrypted data. nGraph-HE incorporates a privacy-preserving abstraction layer, enabling HE-aware optimizations at compile- and run-time. The framework supports BFV and CKKS cryptosystems without bootstrapping. For experimental analysis, they use CKKS on fixed-precision numbers as the underlying encryption scheme over an Xeon Platinum 8180 platform with 112 CPUs and 376GB of RAM.

Jiang et al. [59] propose a method to perform arithmetic operations over homomorphically encrypted matrices. They introduce the E2DM framework to demonstrate the applicability of the proposed matrix computation approach for the secure evaluation of NN-HE models. They also considered packing multiple matrices (images) into a single ciphertext and computing on them in a SIMD manner, yielding better-amortized performance; their CryptoNets implementation on MNIST takes 1.69 seconds to compute ten likelihoods of 64 input images simultaneously. E2DM reports the same accuracy for plaintext and ciphertext inference, considering CKKS as the underlying cryptosystem with a security level of 80 bits; however, it remains unclear how a square activation function in an approximate homomorphic computation achieves a zero-precision loss.

Badawi et al. [19] present an efficient NN-HE for image classification on GPUs. The authors use a GPU-based BFV implementation as an underlying engine to perform encrypted computations. The heterogeneous GPU cluster consists of three Nvidia Tesla P100 cards with 3584 CUDA cores and one Nvidia V100 with 5120 cores. The MNIST-HCNN model is five layers deep: two sequential convolutional layers with their respective square activation function followed by one dense layer. For CIFAR-10, they provide an 11-layer network: three convolutional layers, each one followed by a square activation and HE-friendly pooling layer, and finally, two dense layers. The HCNN model significantly accelerates the classification process while maintaining security and accuracy; it classifies MNIST in 2% of the time CryptoNets takes.

Falcetta and Roveri [60] develop a privacy-preserving LeNet-1 model variant by incorporating some of the best practices in the NN-HE field, such as replacing non-HE-compliant activation functions with low-degree polynomials, max pooling with average pooling, etc. They highlight the security parameter selection and the approximation of non-linear layers as the primary considerations in constructing secure and accurate NN-HEs.

The main challenge toward deploying HE schemes in real-world applications lies in overcoming the high computational costs associated with these cryptosystems. Computing over ciphertexts with state-of-the-art schemes such as CKKS represents a slowdown of 4-6 orders of magnitude compared to performing the same computations on unencrypted data [64]. In recent years, several researchers have supported the evaluation of complex DNN models over encrypted data. We remark that the highest state-of-the-art DNN accuracy on MNIST and CIFAR-10 in the unencrypted domain is 99.79% and 96.53%, respectively.

Lee et al. [50] implement a privacy-preserving ResNet-20 model using the Residue Number System CKKS (RNS-CKKS) scheme with bootstrapping. It is based on the theoretical contribution presented in [65], where authors propose a precise polynomial approximation technique for the ReLU and max-pooling functions using a composition of minimax approximate polynomials of a small degree. Lee et al. [50] homomorphically evaluate the NN-HE with 383 CIFAR-10 images and plaintext model parameters. Nonetheless, the proposed model takes about 3 hours to infer one image due to the more than a thousand bootstrapping functions implemented and the high-degree minimax composite polynomials used for approximating ReLU and Softmax functions. Although such latency is high for practical use, it represents a significant step toward a DNN with FHE using bootstrapping.

Pure MPC and hybrid MPC-HE-based solutions, such as presented in [43, 66–73], are outside the scope of the current study and, therefore, are omitted. For instance, in an MPC-HE approach like [43], the client carries out the bootstrapping process directly.

Privacy-preserving NN models have come a long way from the first introduction to actual solutions. Several companies, such as IBM [74] and Microsoft [75], offer HE services and incorporate HE into their commercial privacy-aware solutions. However, there is still a long way to go and daunting challenges. We are observing a developing research area in constant growth, with many potential applications and significant privacy benefits.

Table 2 summarizes the polynomial approximation methods used in the state-of-the-art NN-HEs to address cryptographically non-computable activation functions: Square function, Taylor series, Least-squares, Chebyshev polynomials, Newton, and Composite polynomials, implemented in CryptoNets, Chabanne-NN, Faster-CryptoNets, SEALion, Liao-CNN, CryptoDL, Lo-La, nGraph-HE, E2DM, HCNN, LeNet-HE, RNS-CKKS-NN, and our CNN-HE-SLAF model.

**Table 2:** Summary of activation function approximations used in state-of-the-art privacy-preserving neural network models with homomorphic encryption.

Year	Model	Function approximated	Method	Degree	Ref
2016	CryptoNets	ReLU	Square function	2	[54]
2017	Chabanne-NN	ReLU	Taylor series, Least-squares	2–6	[47]
2018	Faster-CryptoNets	ReLU, Swish, Softplus	Chebyshev approximation	2	[44]
2019	SEALion	ReLU	Square function	2	[56]
2019	Liao-CNN	ReLU, Swish, Tanh	Chebyshev series	3–6	[63]
2019	CryptoDL	ReLU, Sigmoid, Tanh	Chebyshev series	2–8	[42]
2019	Lo-La	ReLU	Square function	2	[57]
2019	nGraph-HE	ReLU	Chebyshev approximation	2	[58]
2019	E2DM	ReLU	Square function	2	[59]
2020	HCNN	ReLU	Square function	2	[19]
2022	LeNet-HE	ReLU	Square function	2	[60]
2022	RNS-CKKS-NN	ReLU	Newton, Composite polynomials	7, 15, 27	[50]
2023	CNN-HE-SLAF	ReLU	Self-learning polynomials	3	

## 6. Related work

In recent years, there has been a growing interest among the research community in exploring non-homomorphic activation functions that are trained during the learning process. In this section, we review the latest advances in this emerging field. First, we examine the taxonomy of trainable activations. Later, we highlight related works in the field of adaptative polynomial activation functions.

The idea behind a trainable activation is to search for an adequate function shape using knowledge from the training data [76]. There are three main adaptive activation families [77]: parametrized activations, ensemble-based activations, and trainable polynomial activations [78].

The first family refers to those parametrized activations such as PReLU [79], PELU [80], RePU [81], Swish [82], and Syncular [83], which are based on conventional activation functions and fine-tuned through the incorporation of one or more trainable parameters.

The second family includes activations based on ensemble methods [84–86]. These approaches comprise a collection of basic functions, which can consist of standard activations, trainable functions, or a combination of both, and a model that defines the linear combination of such a basis.

The third family refers to those polynomial activation functions with trainable coefficients, where the polynomial coefficients are learned together with the network parameters using the backpropagation learning algorithm and gradient descent optimization method.

The first and second families involve non-polynomial functions and use operations not supported by HE cryptosystems. Therefore, we focus on the third family of trainable activation functions, which consists of HE-friendly polynomials with coefficients that can be effectively learned and adjusted.

Hou et al. [78] propose a piecewise polynomial Smooth Adaptive Activation Function (SAAF) on non-homomorphic CNN models for regression tasks. The parameters of SAAF control the shape of activation functions, where these parameters are trained along with other NN model parameters. The authors argue that applying SAAFs in the regression (second-to-last) layer of a NN can significantly decrease the bias of the regression NN and avoid overfitting by simply regularizing the model. They report results over eight real-world datasets while incurring a minimal increase of less than 1% in the total number of model parameters.

Goyal et al. [87] introduce Self-Learning Activation Functions (SLAF) over non-homomorphic NN models. SLAFs are learned during training and can approximate most existing activation functions. SLAFs are a weighted sum of Taylor polynomials that can accurately approximate the optimal activation. According to the authors, non-homomorphic NN models with SLAF improve the accuracy concerning equivalent architectures with conventional activation functions on standard benchmarking datasets.

In the third family, special attention is given to trainable rational activation functions. These functions are defined as the ratio of two polynomials *P*(*x*) and *Q*(*x*), both with trainable coefficients and respective degrees *m* and *n*. Rational functions are better suited than polynomials to approximate ReLU [88]. They demonstrate competitive accuracy on non-homomorphic NN models for image classification [89–91]. Additionally, trainable Padé activations can approximate standard hand-designed activations and learn new activations with compact representations [89]. Nevertheless, their inherent inverse operation makes it infeasible for encrypted computation.

Several studies indicate that NNs with polynomial activations have the same representational power as their non-polynomial counterparts [92, 93]. Additionally, these polynomial activations enhance latency without compromising accuracy [62].

While multiple works in the NN-HE field have explored using polynomial activation functions to enable an efficient homomorphic classification (see Table 2), trainable polynomial activation functions remain unexplored. We propose the privacy-preserving SLAF-based NN-HE (NN-HE-SLAF) model, introducing a novel approach in the encrypted classification domain by considering self-learning polynomial approximations to generate cryptographically computable activation functions. We use a customized polynomial with trainable coefficients as an activation function. In contrast to the traditional use of a single fixed-interval approximation of a specific activation function for all neurons, NN-HE-SLAF models generate customized polynomials for each homomorphic neuron independently, yielding better accuracy and performance.

## 7. Self-learning activation functions

In this section, we present SLAF models for designing a privacy-preserving NN-HE. Given the definition of a homomorphic neuron of the NN-HE model by Eq (5), we approximate the non-linear activation function by a polynomial at each neuron independently with trainable coefficients as

$$\ddot{f_{k}}=a_{0}^{k}+a_{1}^{k}x+a_{2}^{k}x^{2}+\cdots +a_{n}^{k}x^{n}$$ (6)

where $a_{0}^{k},a_{1}^{k},\ldots ,a_{n}^{k}$ denote the trainable coefficients of the polynomial $\ddot{f_{k}}$ at neuron *k*.

The central concept of the SLAF training process is to find an adequate mapping from the input to the output space based on modifying NN weights and polynomial coefficients in each neuron separately. NNs can learn and generalize more information based on training examples. The process is an analogy of the human brain evolution during a person’s experience.

In addition to synaptic weights, the training process aims to find the SLAF polynomial coefficients. Therefore, NN-HE-SLAF activations are adapted to the specific problem, structure of the network, its parameters, and the dataset.

**Fig 1:**
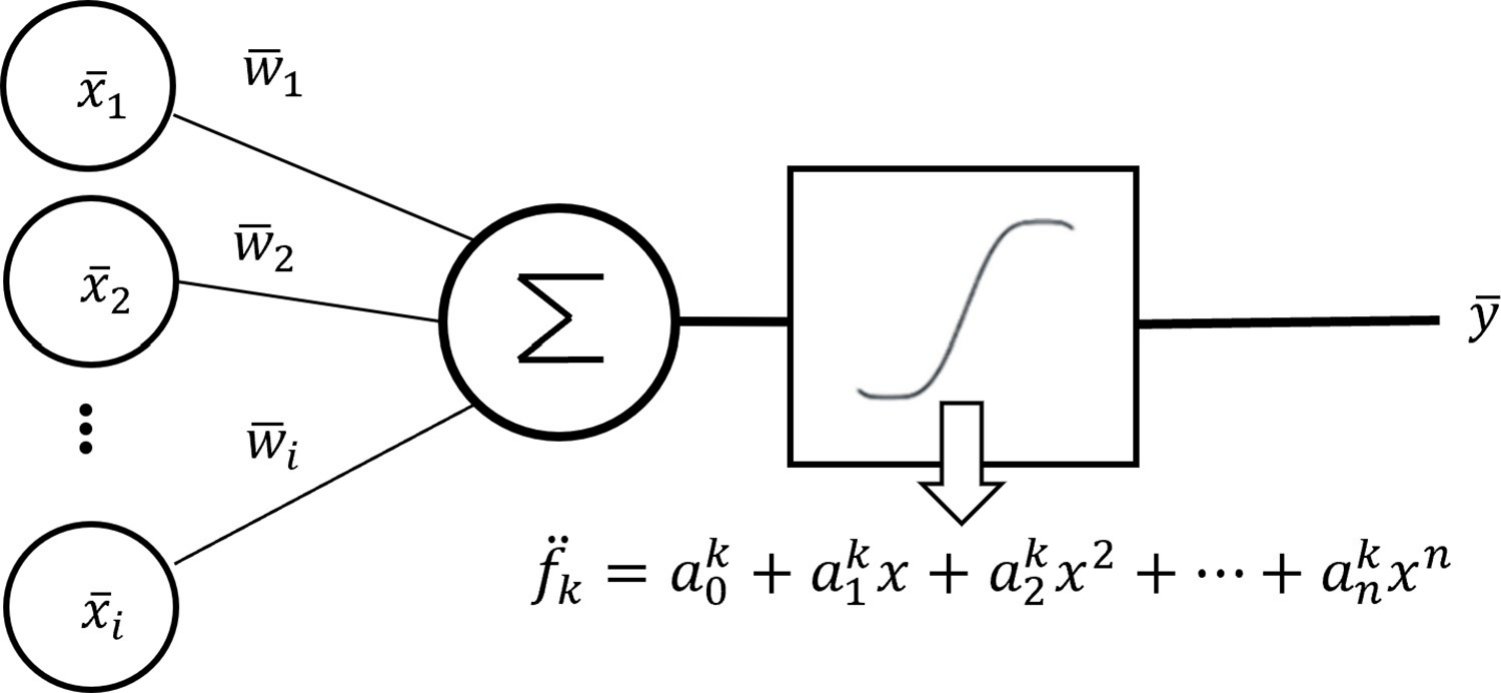
Homomorphic neuron *k* with a self-learning activation function ${\ddot{f}}_{k}$.

Fig 1 shows the general structure of a homomorphic neuron with SLAF.

We study two approaches for polynomial initialization: SLAF_(0)_ and SLAF_(P)_. SLAF_(0)_ considers *n*+1 trainable polynomial coefficients *a*_0_, *a*_1_,…,*a*_*n*_ initialized by zero. In the SLAF_(P)_ approach, coefficients are initialized by coefficients obtained by known approximation methods. While SLAF_(0)_ starts searching from the “zero point” of the solution space, SLAF_(P)_ explores a search space close to the best approximation solution.

Let us consider an example of SLAF_(P)_ using as a polynomial the nine-degree approximation *f*_*c*_ of the *sign*(*x*) function performed by the composition of minimax approximate polynomials [21].

**Fig 2:**
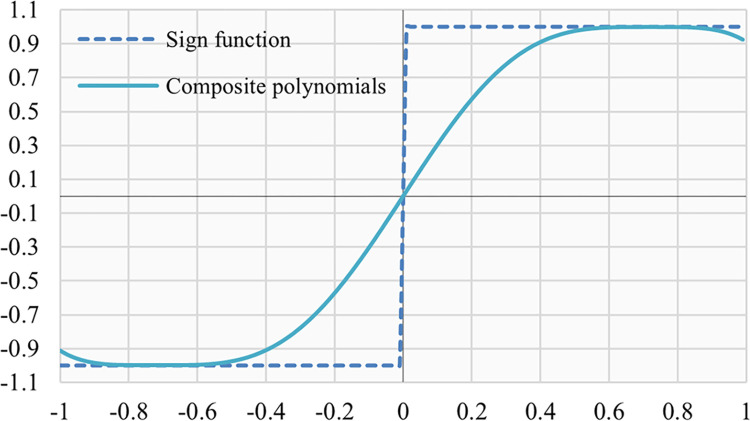
Nine-degree polynomial approximation of the sign function generated by composite polynomials of the minimax approximate polynomials.

Fig 2 shows the approximation of the *sign*(*x*) function given by

$$f_{c}=3.114x-6.645x^{3}+8.851x^{5}-5.485x^{7}+1.169x^{9}$$ (7)

SLAF_(P)_ polynomial is initialized by ten coefficients *γ*_0_ = 0, *γ*_1_ = 3.114, *γ*_2_ = 0, *γ*_3_ = −6.645, *γ*_4_ = 0, *γ*_5_ = 8.851, *γ*_6_ = 0, *γ*_7_ = −5.485, *γ*_8_ = 0, and *γ*_9_ = 1.169. The training results in new polynomial coefficients *γ*_0_, *γ*_1_,…,*γ*_*n*_, that is

$$\ddot{f_{k}}=\gamma_{0}^{k}+\gamma_{1}^{k}∙x+\gamma_{2}^{k}∙x^{2}+\cdots +\gamma_{n}^{k}∙x^{n}$$ (8)

where $\gamma_{0}^{k},\gamma_{1}^{k},\ldots ,\gamma_{n}^{k}$ denote the trained SLAF_(P)_ coefficients at neuron *k*. All neurons initially have identical *γ*_0_, *γ*_1_,…,*γ*_*n*_, but post-training exhibits unique coefficients $\gamma_{0}^{k},\gamma_{1}^{k},\ldots ,\gamma_{n}^{k}$ for each neuron *k*.

Considering that an NN mimics the behavior of the biological brain, SLAF refers to the physiological capacity of the human brain called plasticity. This ability allows the brain to adapt to new learning methods by conditioning and remodeling how neurons connect. Our NN-HE-SLAF models not only enable the identification of underlying relationships in the information but also facilitate the determination of specific activation functions for a given dataset that guide the learning.

To better understand the capabilities of SLAF, we prove its feasibility in approximating a continuous activation function.

**Theorem 1.** An NN-HE-SLAF can approximate any continuous activation function *f* if its input domain is bounded to a desired error as a function of the SLAF degree.

### Proof

On the one hand, the Universal approximation theorem, also known as the Hornik theorem, establishes that every continuous function can be approximated by an NN with at least a single hidden layer with arbitrary precision concerning an *L*_*p*_-norm. Since a polynomial is a continuous function, it can be approximated by a NN.

On the other hand, the Stone-Weierstrass theorem establishes that every continuous function can be approximated by a polynomial in a bounded interval. Specifically, for any continuous and real-valued function *f*(*x*) defined on the interval [*a*, *b*], for every *δ*>0, there exists polynomial *p*(*x*) s.t. for ∀*x*∈[*a*, *b*], we have

|f(x)−p(x)|<δ (9)

Since the input domain is restricted, the bounded property holds even if the activation function is unbounded, e.g., ReLU, PELU, SeLU, ELU, etc. Moreover, if *f*(*x*) is not a polynomial, the degree of *p*(*x*) tends to infinity as *δ* approaches zero.

From the Weierstrass theorem, it follows that continuous activation functions, such as ReLU, Swish, etc., can be approximated by a polynomial. Then, such activations can be approximated by an NN-HE-SLAF with at least a single dense layer. *The theorem is proved*.

## 8. Case study

This section presents details of the proposed models. To provide a fair comparison, we implement NNs with a similar architecture to the ones used in state-of-the-art models, such as CryptoNets [54], Faster-CryptoNets [44], and HCNN [19] (see Table 1).

We study two SLAF models based on CNN: CNN-HE-SLAF with a single training phase and CNN-HE-SLAF-R with training and re-training.

In the first model, we replace all activation functions with three-degree SLAF_(0)_ or SLAF_(P)_ and train CNN to find weights and coefficients. We denote these models as CNN-SLAF_(0)_ and CNN-SLAF_(P)_.

In the second model, we train CNN with original non-homomorphic activations function, lock weights, substitute activation functions by polynomials SLAF_(0)_ and SLAF_(P)_, and (R)e-train CNN to adapt coefficients. We denote these models as CNN-SLAF_(0)_-R and CNN-SLAF_(P)_-R, respectively.

**Fig 3:**
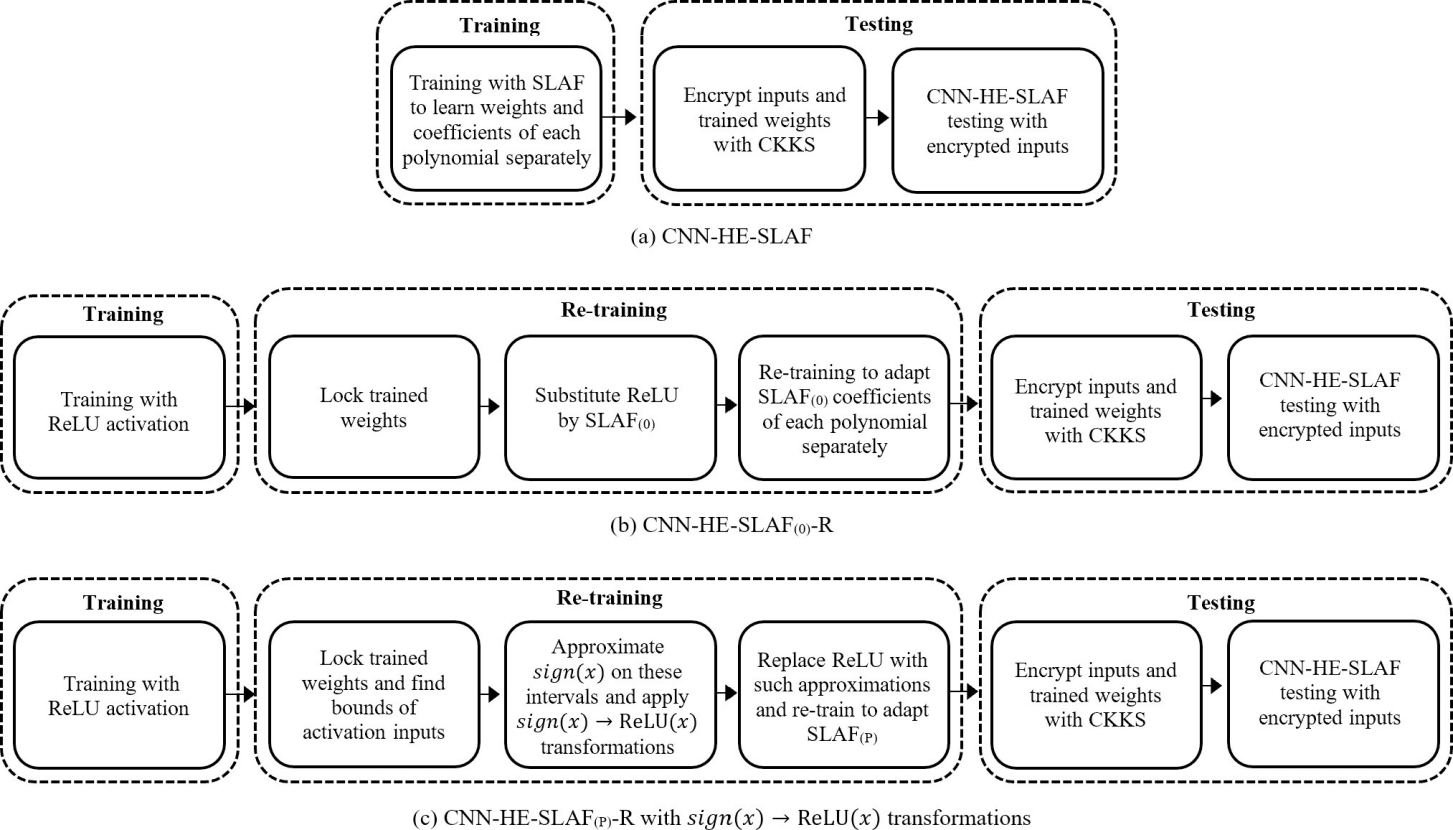
Training and testing privacy-preserving CNN-HE models with self-learning activation functions.

Fig 3 illustrates the training and testing procedures for the proposed privacy-preserving CNN-HE-SLAF, CNN-HE-SLAF_(0)_-R, and CNN-HE-SLAF_(P)_-R models.

**Fig 4:**
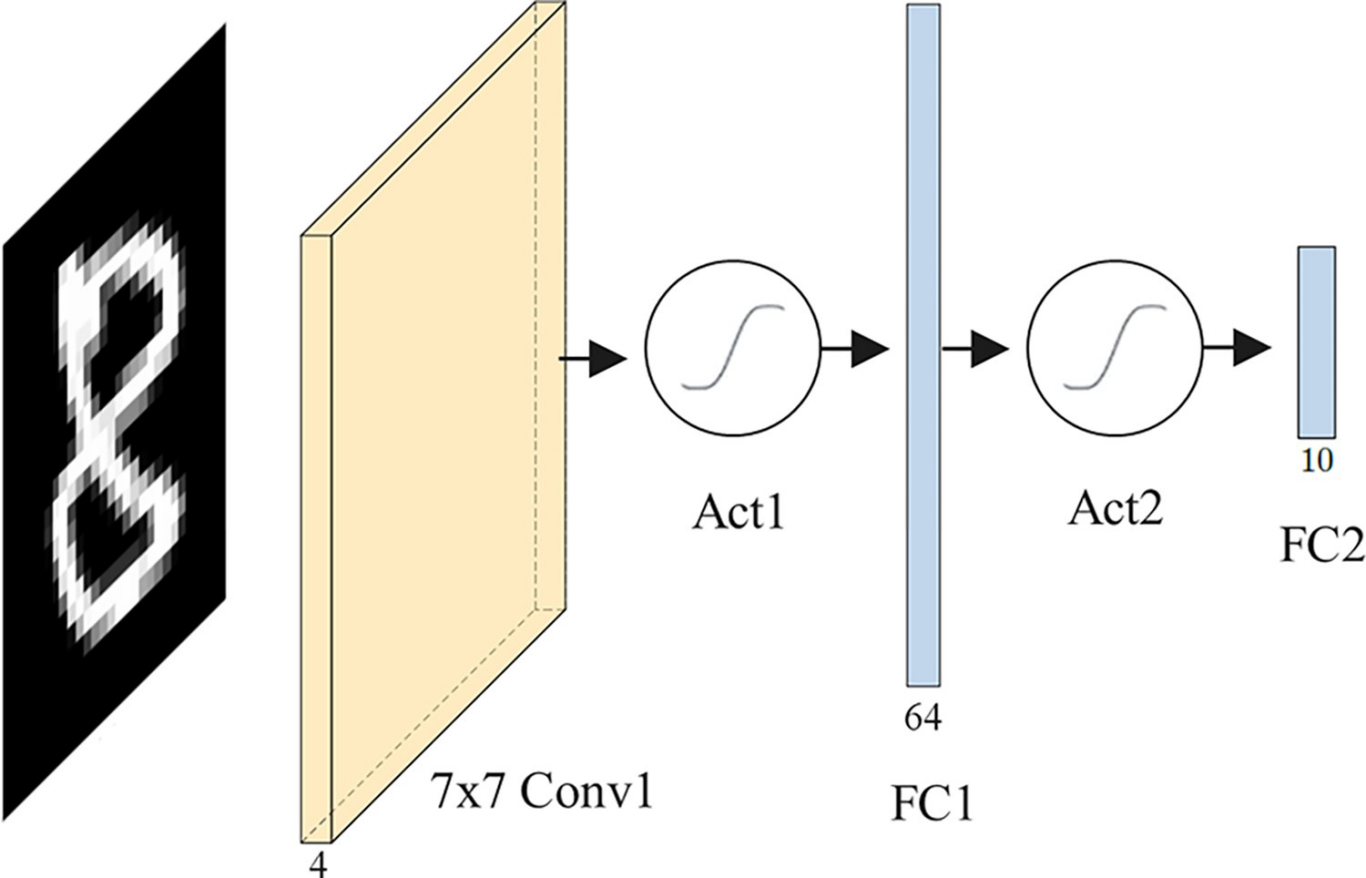
CNN1 with a single convolutional layer. The yellow element denotes a convolutional layer. Circular elements after Conv1 and FC1 denote activation functions. Blue elements represent dense layers.

**Fig 5:**
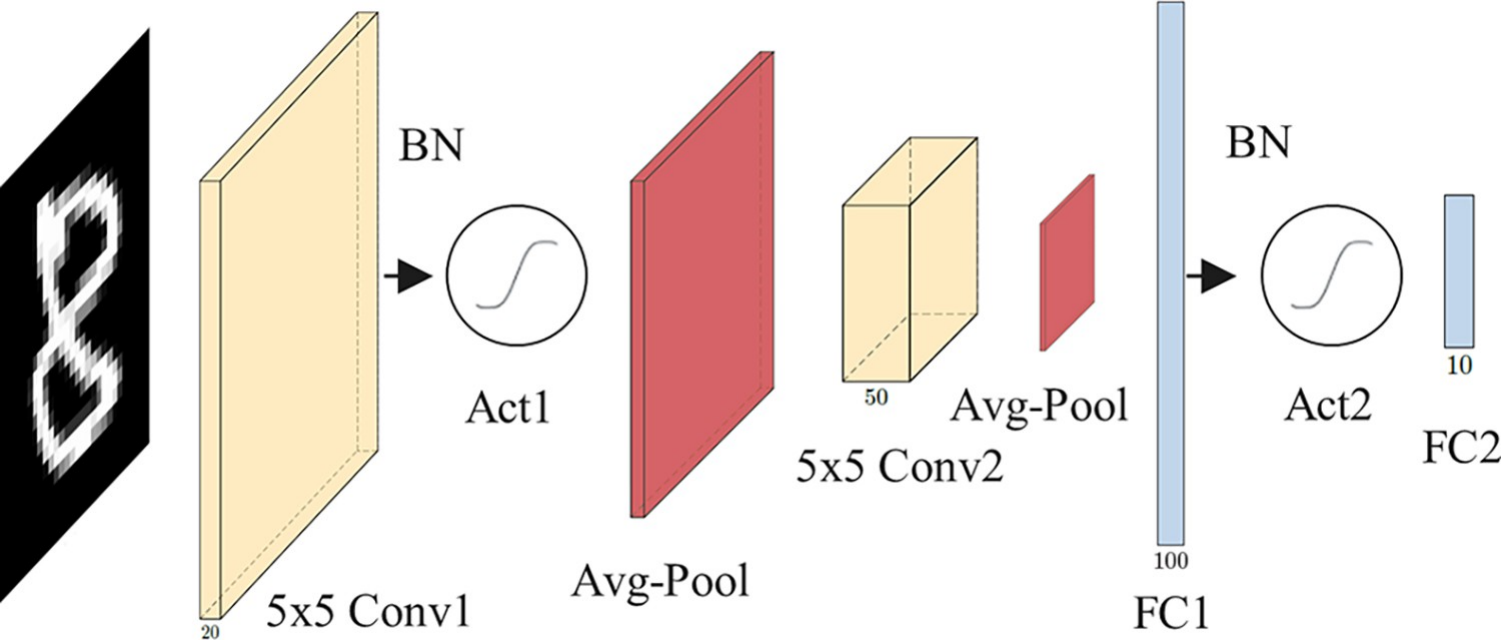
CNN2, a CryptoNets-based network with two convolutional layers. Red elements denote average pooling layers. Circular elements after Conv1 and FC1 denote activation functions. Blue elements represent fully connected layers.

We study two architectures of CNNs: 1-convolutional (CNN1) and 2-convolutional (CNN2). Figs 4 and 5 illustrate their structures.

CNN1, with a single convolutional layer and two fully connected layers, is a variant of Lo-La small NN [57]. In contrast to the Lo-La approach, CNN1 incorporates approximated activations after the convolutional and the first fully connected layers. CNN1 details are presented in Table 3.

**Table 3:** CNN1 architecture.

Layer	Description	Output shape
Input	The image has a size of 28x28 pixels.	1 x 28 x 28
Convolution	4 filters of size 7x7, stride (3,3) without padding.	4 x 8 x 8
Activation	The approximate activation function is applied to each input value.	4 x 8 x 8
Fully connected	A weighted sum of the entire previous layer with 64 filters.	64
Activation	The approximate activation function is applied to each input value.	64
Fully connected	Generates 10 outputs (corresponding to the 10 digits) from the 64 inputs.	10

CNN2 is a CryptoNets-based network architecture with two convolutional layers. To provide a direct comparison concerning state-of-the-art solutions, we adopt the architecture proposed in [44], which in turn is a variant of CryptoNets, CryptoDL, Lo-La, HCNN, SEALion, and others. It incorporates a BN layer before each activation to encourage the activation inputs to fit in the approximated interval.

The BN layer transforms them into normal distribution inputs with zero mean and unit variance, which reduces the overall approximation error, prevents the generalization of higher feature values and indirectly provides smaller weights for homomorphic processing. The details of such an architecture are presented in Table 4.

**Table 4:** CNN2 architecture.

Layer	Description	Output shape
Input	The image has a size of 28x28 pixels.	1 x 28 x 28
Convolution	20 filters of size 5x5, stride (2,2), and padding of 1.	20 x 13 x 13
Batch normalization	Batch normalization is applied to each input value.	20 x 13 x 13
Activation	The approximate activation function is applied to each input value.	20 x 13 x 13
Pooling	Average pooling with windows 3, stride 2, and padding of 1.	20 x 7 x 7
Convolution	50 filters of size 20 x 5 x 5 and stride (1, 1) without padding	50 x 3 x 3
Pooling	Average pooling with windows 3, stride 2, and padding of 1.	50 x 2 x 2
Fully connected	A weighted sum of the entire previous layer with 100 filters.	100
Batch normalization	Batch normalization is applied to each input value.	100
Activation	The approximate activation function is applied to each input value.	100
Fully connected	Generates 10 outputs from the 100 inputs.	10

For CNN1 and CNN2 with SLAF_(0)_-R and SLAF_(P)_-R, we use the original ReLU function in the first training. Then, we lock trained weights, replace ReLU activation functions with SLAF_(0)_ or SLAF_(P)_ approximations, and re-train CNN to learn customized polynomial approximation coefficients. For re-training, we choose considerably fewer epochs than for training (see Section 9.3: Developing tools).

Several state-of-the-art models, as presented in [40, 50, 65], have proposed high-degree approximations to obtain an accuracy similar to ReLU. They state that a precise approximation of the ReLU function is necessary to evaluate models applied to plaintext or encrypted inputs.

**Fig 6:**
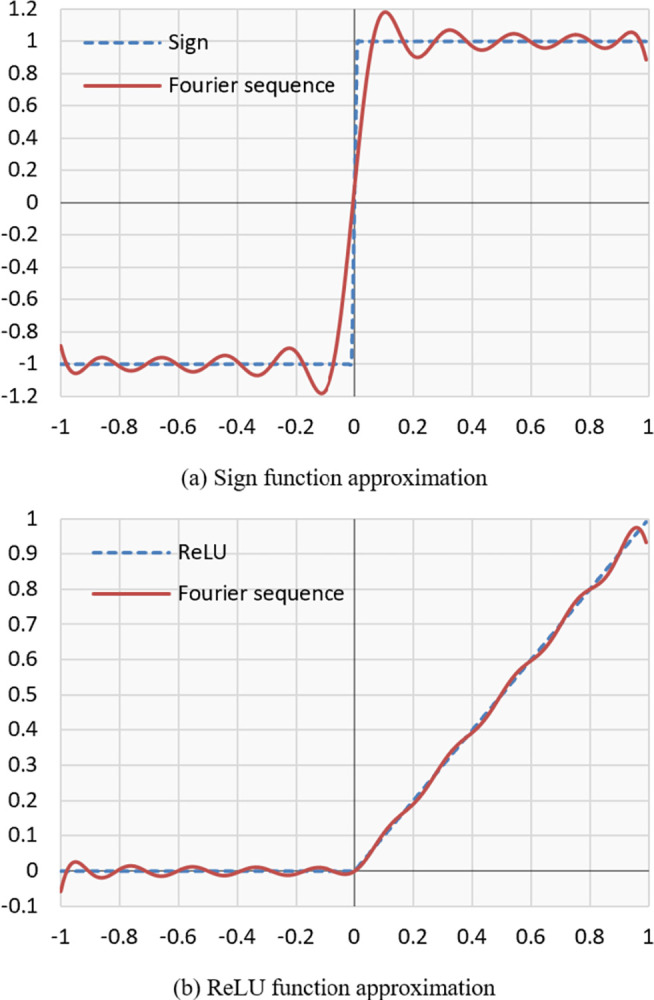
Polynomial approximation of sign function on the interval [−*B*, *B*] with *B* = 1 and their corresponding ReLU approximation based on the equivalence *ReLU*(*x*)≈[*x*+*x*∙*sign*(*x*)]/2.

To construct a better ReLU approximation for each neuron independently, we collect data during the first training to find intervals of activation inputs. Then, we generate a polynomial approximation of *sign*(*x*) function on found symmetric intervals [−*B*, *B*], where *B* denotes the supremum norm of the activation inputs of each neuron. Due ReLU(*x*) is equivalent to $\frac{x+|x|}{2}$ and |*x*| = *x*∙*sign*(*x*), we approximate $ReLU(x)\approx \frac{x+x∙sign(x)}{2}$ (see Fig 6). A large *B* has the advantage of enlarging the input ranges of the polynomial activation function at the cost of a higher approximation error.

For approximation methods such as Chebyshev polynomials, which are orthogonal in [−1, 1], the interval is extended from [−1, 1] to [−*B*, *B*] by *p*(*x*/*B*) for *B*>1. Similarly, for Newton-Schulz, which approximates the ±1 roots of $f(x)=1-\frac{1}{x^{2}}$ by the iterative computation of $f(x)=\frac{x}{2}∙(3-x^{2})$, converging to *sign*(*x*) in [−1, 1].

Fig 6 shows the polynomial approximation of the sign function by a Fourier sequence (Fig 6A) and its respective ReLU approximation based on the equivalence $ReLU(x)\approx \frac{x+x∙sign(x)}{2}$ (Fig 6B).

We can see that this simple transformation using *sign*(*x*) approximation allows better ReLU approximation.

## 9. Experimental setup

This section describes the evaluation method and dataset characteristics and defines the experimental setup.

### 9.1. Security settings

The ciphertext size, scheme performance, multiplicative depth, and security level depend on the security parameter settings. We adopt the security settings specified in the HE standard [94]. Table 5 shows such security settings for the CKKS scheme. The security level λ = 128 bits guarantees that an adversary needs to perform 2^128^ elementary operations to break the scheme with a probability one.

**Table 5:** Security settings for the CKKS scheme.

Parameter	Security settings	
	SLAF_(0)_	SLAF_(P)_
*λ*	128	128
*N*	2^14^	2^14^
Δ	2^26^	2^25^
log *q*	366	380
*L*	13	16
*q*	[40, 26,…,26, 40]	[40, 25,…,25, 40]

### 9.2. Dataset

The Modified National Institute of Standards and Technology (MNIST) database is a standard dataset widely used in the literature [95]. It consists of 60,000 grayscale images of handwritten digits. Each image is a 28x28 pixel array, where the value of each pixel is a positive integer in the range [0, 255]. The MNIST training set includes 50,000 examples. The remaining 10,000 images represent the testing set. While MNIST is arguably a simple dataset, it has remained the standard benchmark for homomorphic inference tasks [42, 65].

### 9.3. Developing tools

The HE schemes, homomorphic operations, and NN-HE models are implemented using PyTorch [96] and the open-source Simple Encrypted Arithmetic Library (SEAL) v3.5.6 [97] through the Python TenSEAL library [98].

The experimental evaluation is performed on a server Express x3650 M4 with Intel(R) Xeon(R) CPU E5-2650v2 95W at 2.6GHz, 64 GB. The 64-bit server OS is Ubuntu 18.04.6.

To understand the generalization capacity of the proposed solutions and avoid overfitting, we vary the number of epochs for training in the range of 2-50 and utilize a batch size of 64 and a cross-entropy loss function. The networks are trained with Stochastic Gradient Descent (SGD) with a momentum of 0.9. 30 epochs better train CNN1 and CNN2 for testing accuracy.

For re-training, we consider from 2 to 20 epochs. For CNN-SLAF_(0)_-R with Adam optimizer and a learning rate of 0.001, 5 epochs provide better testing accuracy. For CNN-SLAF_(P)_-R with SGD, 12 epochs provide better testing accuracy.

The learning rate is the most important hyper-parameter to tune for training NN models [99]. We apply the 1-cycle policy [100], also called the super-convergence phenomenon. It uses one round of an increasing and decreasing learning rate, in which the maximum learning rate serves as a regularizer. Simplified activation may not provide a good approximation. A highly flexible activation can lead to overfitting. Since SLAF includes a weighted sum of monomials with trainable weights, employing an effective coefficient regularization strategy is necessary to mitigate the risk of overfitting [78, 87]. The 1-cycle policy enables learning a flexible and accurate activation without compromising the overfitting risk.

We adopt the weight initialization proposed by He et al. [79] for convolutional layers without dropout to obtain the initial synaptic weights. The source code used in this study has been made openly available to the scientific community as a public repository.

## 10. Experimental analysis

To compare traditional CNNs and solutions with SLAF, we analyze their differences and similarities in the training and testing phases. We provide the performance evaluation of non-homomorphic CNN1 and CNN2 on plaintext inputs and privacy-preserving CNN1-HE and CNN2-HE on ciphertext inputs. For CNN-HE models, both inputs and weights are encrypted before testing. We perform the following experiments:

First, we train and test non-homomorphic models with the ReLU activation function. The training accuracy of CNN1 and CNN2 with ReLU over plaintext is 99.562% and 99.748%, respectively. The testing accuracy of CNN1 and CNN2 with ReLU over plaintext is 98.56% and 99.38%, respectively.

Second, we evaluate the performance of CNNs and CNN-HEs using a single training to determine both weights and SLAF coefficients.

Third, we analyze CNNs and CNN-HEs by training with ReLU activations. Then weights are fixed, ReLU is replaced by SLAF, and the model is re-trained to adapt SLAF coefficients at each polynomial independently.

Fourth, we evaluate CNNs and CNN-HEs using ReLU in training. We determine the bounded intervals of each neuron’s activation inputs and approximate *sign*(*x*) on these intervals using known polynomial approximations. Then, we apply *sign*(*x*)→ReLU(*x*) transformations and replace ReLU with such approximations. Finally, we perform a re-training process to optimize SLAF coefficients.

### 10.1 CNN1

We provide the performance evaluation of non-homomorphic CNN1 and CNN1-HE without and with SLAF.

Table 6 presents the latency (Lat) and accuracy (Acc) of the CNN1 model using SLAF_(0)_, SLAF_(0)_-R, different polynomials for SLAF_(P)_-R, and Lo-La. Results for plaintext and ciphertext inputs are reported in the CNN1 and CNN1-HE columns, respectively. Latency corresponds to the time required to process a single classification request. *ρ* denotes the generalization gap, which measures the difference between the training and testing accuracies (%). Lo-La corresponds to the original implementation using specifications, parameters, and characteristics proposed in the corresponding paper [57].

**Table 6:** CNN1 performance.

Model	Polynomial	Training Acc. (%)	СNN1			СNN1-HE		
			Lat (s)	Acc (%)	*ρ*	Lat (s)	Acc (%)	*ρ*(%)
Lo-La	-	99.451	0.012	98.05	1.401	0.34	96.92	2.531
CNN1-SLAF_(0)_	-	99.602	0.013	98.12	1.482	3.56	97.87	1.732
CNN1-SLAF_(0)_-R	-	99.442	0.013	98.34	1.102	3.13	**98.22**	1.222
CNN1-SLAF_(P)_-R	Least-squares	99.955	0.013	98.45	1.505	5.75	97.31	2.645
	Chebyshev	99.935	0.013	**98.56**	1.375	5.47	97.96	1.975
	Newton-Raphson	99.730	0.013	98.55	1.180	5.14	97.56	2.170
	Composition	99.853	0.013	98.55	1.303	5.29	97.86	1.993

We can see that CNN1-SLAF_(P)_-R with three-degree Chebyshev polynomials achieves the same accuracy of 98.56% as the non-homomorphic CNN1 with the original ReLU activation function. CNN1-SLAF_(0)_-R has the best accuracy of 98.22% in CNN-HE models. That is 1.3% better than 96.92% HE Lo-La.

CNN1 and CNN1-HE latencies illustrate the significant overhead of NN-HE models compared with their unencrypted analogous. For example, computing CNN1-SLAF_(0)_-R over ciphertexts results in a slowdown of approximately 240 times compared to performing the same computations on unencrypted data.

To evaluate the effectiveness of SLAF_(P)_ approach over traditional polynomial approximation methods, we test CNN1-HE using known approximations to replace the ReLU activation: Least-squares, Chebyshev polynomials, Newton-Raphson, and Minimax composition. Polynomial approximations to an activation function usually consider fixed approximation intervals regardless of the particular dataset and problem. CNN1-SLAF_(P)_-R with adaptive intervals for each neuron increases the model accuracy.

With a Chebyshev series, CNN1-HE-SLAF_(P)_-R achieves an accuracy of 97.96%, showing an improvement of 11.85% compared to the 86.11% of the Chebyshev series that approximates ReLU without SLAF_(P)_. With composite minimax polynomials, it achieves an accuracy of 97.86%, improving by 18.55% over 79.31% of Composite minimax polynomials without SLAF_(P)_. With Least-squares, it provides an accuracy of 97.31%, improving by 6.01% over 91.30% of Least-squares without SLAF_(P)_. With Newton-Raphson, it has 97.56%, improving by 33.27% over 64.29% of Newton-Raphson without SLAF_(P)_.

### 10.2. CNN2

**Fig 7:**
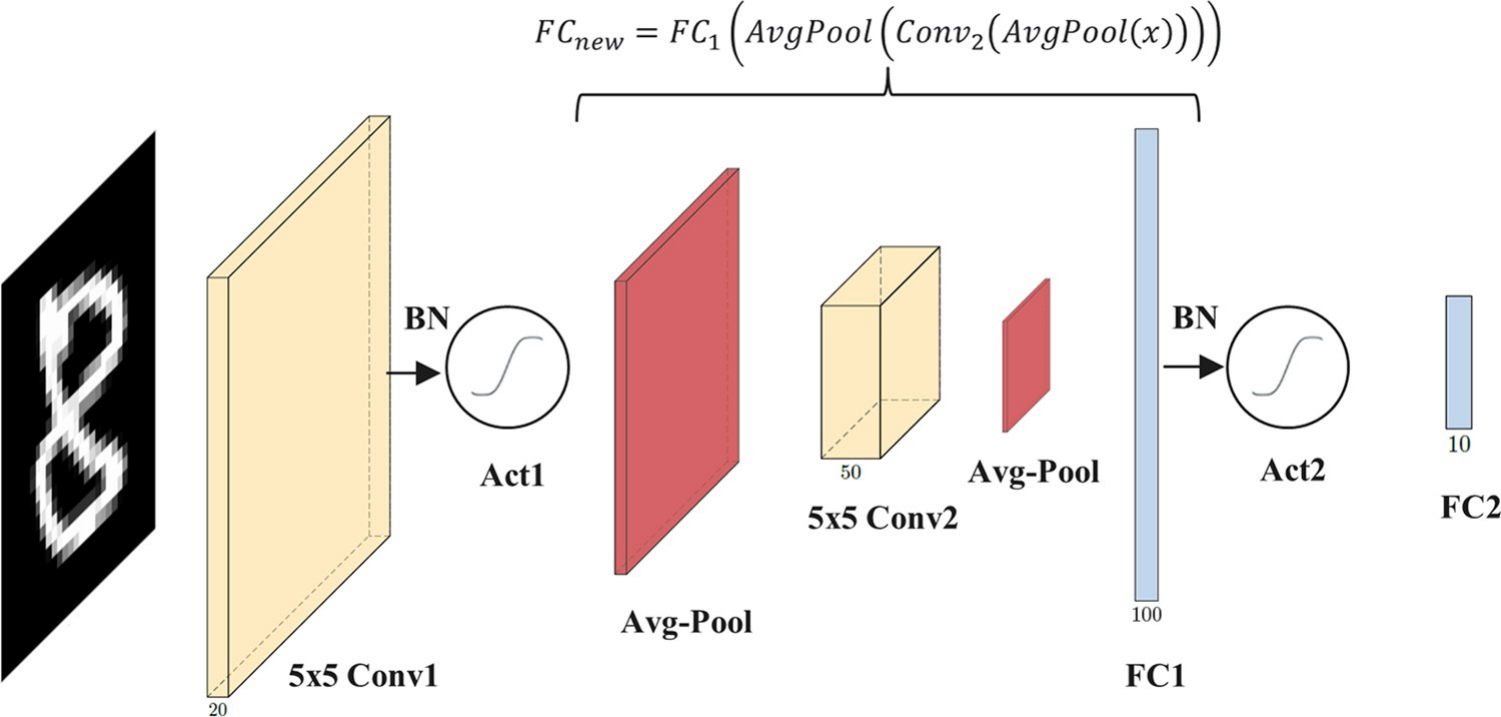
Collapsing CNN2 adjacent linear layers.

Since the combination of linear operations is linear and there is no intervening non-linearity between the first pooling layer and the first fully-connected layer of CNN2, the hidden layers between them can be collapsed into a single linear layer for the testing process, as depicted in Fig 7.

This linear transformation

$$FC_{1}(AvgPool(Conv_{2}(AvgPool(x))))$$ (10)

can be accurately reproduced (up to some numerical rounding error) by the dense layer *FC*_*new*_. Collapsed models exhibit the same accuracy as the original models.

Tables 7 and 8 present the performance of the non-homomorphic and homomorphic CNN2, respectively.

**Table 7:** Non-homomorphic CNN2 performance.

Model	Polynomial	СNN2			
		Training	Testing		
		Acc (%)	Lat (s)	Acc (%)	*ρ*
CNN2-ReLU	-	99.748	0.018	**99.38**	0.368
CNN2-SLAF_(0)_	-	99.907	0.019	**99.38**	0.527
CNN2-SLAF_(0)_-R	-	99.338	0.019	99.32	0.018
CNN2-SLAF_(P)_-R	Least-squares	99.768	0.026	99.35	0.418
	Chebyshev	99.752	0.026	**99.38**	0.372
	Newton	99.645	0.026	99.37	0.275
	Composition	99.712	0.026	**99.38**	0.332

**Table 8:** Performance of privacy-preserving CNN2 model with homomorphic encryption.

Model	Polynomial	СNN2-HE		
		Lat (s)	Acc (%)	*ρ*
CryptoNets	-	250.00	98.95	0.798
F-CryptoNets	-	39.10	98.70	1.048
CryptoDL	-	148.97	98.52	1.228
Lo-La	-	2.20	98.95	0.798
nGraph-HE	-	16.72	98.95	0.798
E2DM	-	**1.69**	98.10	1.648
HCNN	-	5.16	99.00	0.748
CNN2-HE-SLAF_(0)_	-	39.91	98.96	0.947
CNN2-HE-SLAF_(0)_-R	-	39.84	**99.21**	0.128
CNN2-HE-SLAF_(P)_-R	Least-squares	55.97	98.88	0.888
	Chebyshev	55.31	99.05	0.702
	Newton-Raphson	54.21	99.02	0.625
	Composition	55.25	99.05	0.662

CNN2-SLAF_(0)_ and CNN2-SLAF_(P)_ models using low-degree polynomials achieve the same accuracy of 99.38% as the standard ReLU activation function over non-homomorphic models in the plaintext space.

These results show the remarkable capability of SLAF variants to yield comparable accuracy to traditional non-linear functions. It confirms that NN models with SLAF have the same representative ability as their non-polynomial counterparts.

Now, let us evaluate the performance of CNN2-HE-SLAF models in comparison to the state-of-the-art models in the ciphertext space, such as CryptoNets, Faster-CryptoNets, CryptoDL, Lo-La, nGraph-HE, E2DM, and HCNN. These models adopt the 2-convolutional CryptoNets architecture and implement diverse polynomial approximation methods of ReLU.

As shown in Table 8, the CNN2-HE-SLAF_(0)_-R (CNN2-HE-SLAF_(P)_-R) model with re-training achieves an accuracy of 99.21% (99.05%), showing improvements between 0.21% (0.05%) and 1.11% (0.95%) over CryptoNets-based NN-HE solutions, 0.21% (0.05%) over 99% of HCNN, 0.26% (0.10%) over 98.95% of CryptoNets, 0.51% (0.35%) over 98.70% of Faster-CryptoNets, 0.69% (0.53%) over 98.52% of CryptoDL, and 1.11% (0.95%) over 98.10% of E2DM.

CNN2-HE-SLAF_(0)_-R (CNN2-HE-SLAF_(P)_-R) also outperforms models with a similar 2-convolutional architecture, such as LeNet-HE and SEALion (see Table 1), showing accuracy improvements of 1.03% (0.87%) over 98.18% of LeNet-HE and 0.30% (0.14%) over 98.91% of SEALion, respectively. Furthermore, the CNN2-HE-SLAF_(0)_-R (CNN2-HE-SLAF_(P)_-R) improves accuracy by 0.61% (0.45%) compared to 98.60% of the TAPAS model, which utilizes a 19-layer architecture with six convolutional layers.

Comparing performance, we demonstrate that using trainable three-degree polynomial activations, CNN2-HE-SLAF_(0)_-R classifies MNIST 6.26 times faster than CryptoNets with better accuracy (99.21% vs 98.95%).

## 11. Discussion and conclusions

Common encryption algorithms such as Advanced Encryption Standard (AES) successfully protect stored and transmitted data, preventing third parties and the public from reading them. However, data processing needs a decryption, falling into the problem of data vulnerability. Data have to be searched, computed and analyzed securely. HE is a solution to address the processing of confidential data while it remains encrypted. However, HE performs only addition and multiplication operations efficiently on ciphertexts, increasing impracticality due to the significant computational overhead. This overhead is the biggest challenge for widespread adoption. Optimization techniques are required to improve performance and usability in various domains such as cloud computing, machine learning, healthcare, finance, voting, etc.

NN-HE is a rapidly growing research area with many potential applications and significant benefits. The main direction of the current NN-HE development is to increase accuracy and efficiency. While protecting sensitive data’s privacy is essential, they must also offer acceptable accuracy and time complexity.

The CNN-HE-SLAF solutions contribute to these two goals and existing knowledge by designing an adaptive NN-HE.

This paper proposes methods of CNN-HE optimization by Self-Learning Activation Functions (SLAF). We theoretically prove that NNs with SLAF can approximate any continuous activation function to any desired error as a function of the degree of SLAF. In contrast to the traditional NN-HE approaches, where the activation functions of all neurons are replaced by a single (best) polynomial approximation, NN-HE-SLAF generates customized polynomials for each homomorphic neuron independently. It yields better accuracy and performance.

Two convolutional NN-HE models are proposed: CNN-HE-SLAF and CNN-HE-SLAF-R.

- CNN-HE-SLAF replaces all activation functions with SLAF, and it is trained to find synaptic weights and coefficients together. Then, CNN is encrypted homomorphically. We denote these models as CNN-HE-SLAF_(0)_ if polynomial coefficients are initialized to zero and CNN-HE-SLAF_(P)_ if coefficients are initialized to a known polynomial approximation.
- CNN-HE-SLAF-R is trained with the original activation function, weights are locked, activation functions are substituted by SLAF_(0)_ or SLAF_(P)_, and it is re-trained to adjust coefficients. We denote these models as CNN-HE-SLAF_(0)_-R and CNN-HE-SLAF_(P)_-R, respectively. For re-training, we choose considerably fewer epochs than for training.
- CNN-HE-SLAF leverages the approximation capabilities of neural networks by optimizing polynomial approximations of individual homomorphic neurons, customizing them for a given problem, dataset, network structure, parameters, and approximation intervals.
- CNN-HE-SLAF_(0)_-R and CNN-HE-SLAF_(P)_-R achieve the same accuracy of 99.38% as a non-polynomial non-homomorphic ReLU applying better approximation of *sign*(*x*) on the found intervals and *sign*(*x*)→ReLU(*x*) transformations.
- CNN-HE-SLAF achieves an accuracy of 99.21%, improving the state-of-the-art CNN-HE solutions on the MNIST optical character recognition benchmark dataset.
- CNN2-HE-SLAF collapses the hidden layers between the first pooling layer and the first fully connected layer into a single linear layer with batch normalization to reduce latency while keeping the same accuracy.
- CNN2-HE-SLAF_(0)_-R increases the performance (6.26 times faster) than the state-of-the-art CNN-HE CryptoNets on the MNIST.
- CNN-HE-SLAF demonstrates the feasibility of applying trained polynomials to generate HE-compliant and problem-specific activation functions, enabling efficient, accurate, and privacy-preserving CNN-HE solutions.

These benefits are possible at the expense of increased CNN-HE model complexity. SLAF adds additional trainable parameters, such as coefficients of polynomial approximations of activation functions, making training time longer. So, it is important to find an adequate initialization point and avoid potential overfitting. In future work, it is essential to apply the state-of-the-art practical methods of NN hardware acceleration to SLAF techniques: highly parallel CPU, GPU, Tensor Processing Unit (TPU), Field Programmable Gate Array (FPGA) or Application-Specific Integrated Circuit (ASIC), etc.

Moreover, it is important to validate the applicability and efficiency of the self-learning approach to other ML models, such as logistic regression, and to other CNN-HE layers, e.g., pooling, batch, etc. Furthermore, SLAF performance can be optimized by introducing a dropout mechanism, whereby coefficients below a specific *∈*-threshold are nullified or converted to zero.

Additionally, while CNN-HE-SLAF models can generate homomorphically computable activation functions, these privacy-preserving models inherit both the strengths and weaknesses of current NN models, where even advanced models trained with state-of-the-art optimization techniques do not provide a practical solution for every problem. The SLAF approach does not guarantee the best approximation for a given function and norm. In future work, it is imperative to enhance the approximation and model accuracy by considering SLAF as a weighted sum of orthogonal polynomials instead of monomials.

Another relevant line of future work involves improving the internal structure of the CNN-HE to increase the performance of homomorphic self-adaptive learning using different HE schemes and other state-of-the-art strategies, e.g., incorporating RNS HE variants to decompose the encrypted inputs into several parts and propagating them homomorphically and independently across the model. In addition to the highly parallelizable property, an RNS-based HE scheme allows for smaller encryption parameters without compromising the scheme security level, i.e., a reduced latency, keeping security and accuracy.

Finally, the list of potential applications is broad due to the strongly applied nature in real-world problems and the significant privacy benefits of CNN-HE-SLAF solutions. In future work, it is essential to explore the applicability of proposed models for sensitive domains such as medical image classification.

## Supporting information

S1 Table Main terminology. (PDF)

S2 Table Acronyms. (PDF)
