# Supplementary material for: Self-learning activation functions to increase accuracy of privacy-preserving Convolutional Neural Networks with homomorphic encryption
Source: PLoS One. 2024 Jul 22;19(7):e0306420. doi: 10.1371/journal.pone.0306420 (PMC11262700; doi:10.1371/journal.pone.0306420)
Supplement: S1 Table — (PDF) [file pone.0306420.s001.pdf]

S1 Table. Main terminology

Term	Description
Bootstrapping	Converts a ciphertext into an “equivalent” refreshed ciphertext that contains less noise than the original allowing unbounded homomorphic computations.
Plaintext	Original unencrypted message.
Ciphertext	Encrypted message.
Polynomial approximation	Approximation of a function using addition and multiplication, such that the result is as close to the actual function as possible.
Homomorphism	Transformation of one set into another that preserves in the second set the relations between elements of the first.
Polynomial	Sum of several terms that contain different powers of the same variable.
Norms	At a higher level, norms are measures of the distance between the original function and their approximations.
Noise	Error injected into encrypting messages.
Ring	Algebraic structure that defines a set of elements closed under addition and multiplication. It forms an abelian group under addition and satisfies associativity for multiplication, with the distributive property linking the two operations.
Field	Commutative ring on which non-zero elements form an abelian group under multiplication.
Fixed-precision number	Numerical representation that uses a predetermined number of digits to represent the decimal part of a real value.
