# Supplementary material for: Self-learning activation functions to increase accuracy of privacy-preserving Convolutional Neural Networks with homomorphic encryption
Source: PLoS One. 2024 Jul 22;19(7):e0306420. doi: 10.1371/journal.pone.0306420 (PMC11262700; doi:10.1371/journal.pone.0306420)
Supplement: S2 Table — (PDF) [file pone.0306420.s002.pdf]

S2 Table. Acronyms

Acronym	Description
HE	Homomorphic Encryption
SHE	Somewhat Homomorphic Encryption
FHE	Fully Homomorphic Encryption
ML	Machine Learning
NN	Neural Network
CNN	Convolutional Neural Network
DNN	Deep Neural Network
DiNN	Discretized Neural Network
BNN	Binary Neural Network
NN-HE	Neural Network with Homomorphic Encryption
AF	Activation Function
SAAF	Smooth Adaptive Activation Function
SLAF	Self-Learning Activation Functions
$SLAF_{(0)}$	SLAF with coefficients of polynomials initialized by zero
$SLAF_{(P)}$	SLAF with coefficients initialized to known Polynomial approximations
SLAF-R	SLAF with Re-training
NN-HE-SLAF	SLAF-based NN-HE
ReLU	Rectified Linear Unit
GELU	Gaussian Error Linear Unit
Tanh	Tangent Hyperbolic
RLWE	Ring Learning with Error
MPC	Multi-Party Computation
FL	Federated Learning
DP	Differential Privacy
FE	Functional Encryption
RNS	Residue Number System
CKKS	Cheon-Kim-Kim-Song HE scheme
BGV	Brakerski-Gentry-Vaikuntanathan HE scheme
BFV	Brakerski/Fan-Vercauteren HE scheme
GSW	Gentry-Sahai-Waters HE scheme
YASHE	Yet Another SHE scheme
HPS	Hoffstein-Pipher-Silverman HE scheme
LTV	López-Tromer-Vaikuntanathan HE scheme
TFHE	Fully Homomorphic Encryption over the Torus
HW	Hamming Weight
SIMD	Single Instruction Multiple Data
FC	Fully Connected layer
BN	Batch Normalization
SGD	Stochastic Gradient Descent
GPU	Graphic Processing Unit
TPU	Tensor Processing Unit
Lo-La	Low-Latency CryptoNets solution
MNIST	Modified National Institute of Standards and Technology database
SEAL	Microsoft Simple Encrypted Arithmetic Library
